# Women’s multiple uses of an overdose prevention technology to mitigate risks and harms within a supportive housing environment: a qualitative study

**DOI:** 10.1186/s12905-021-01196-6

**Published:** 2021-02-02

**Authors:** Geoff Bardwell, Taylor Fleming, Ryan McNeil, Jade Boyd

**Affiliations:** 1 British Columbia Centre on Substance Use, 400-1045 Howe Street, Vancouver, BC V6Z 2A9 Canada; 2 Department of Medicine, University of British Columbia, St. Paul’s Hospital, 608-1081 Burrard Street, Vancouver, BC V6Z 1Y6 Canada; 3 Interdisciplinary Studies Graduate Program, University of British Columbia, 270-2357 Main Mall, Vancouver, BC V6T 1Z4 Canada; 4 Yale School of Medicine, 367 Cedar Street, New Haven, CT 06510 USA

**Keywords:** Opioid overdose, Housing, Women, Sex work, Violence, Mobile technologies

## Abstract

**Background:**

North America is amidst an opioid overdose epidemic. In many settings, particularly Canada, the majority of overdose deaths occur indoors and impact structurally vulnerable people who use drugs alone, making targeted housing-based interventions a priority. Mobile applications have been developed that allow individuals to solicit help to prevent overdose death. We examine the experiences of women residents utilizing an overdose response button technology within a supportive housing environment.

**Methods:**

In October 2019, we conducted semi-structured qualitative interviews with 14 residents of a women-only supportive housing building in an urban setting where the overdose response button technology was installed. Data was analyzed thematically and framed by theories of structural vulnerability.

**Results:**

While participants described the utility and disadvantages of the technology for overdose response, most participants, unexpectedly described alternate adoptions of the technology. Participants used the technology for other emergency situations (e.g., gender-based violence), rather than its intended purpose of overdose response.

**Conclusions:**

Our findings highlight the limitations of current technologies while also demonstrating the clear need for housing-based emergency response interventions that address not just overdose risk but also gender-based violence. These need to be implemented alongside larger strategies to address structural vulnerabilities and provide greater agency to marginalized women who use drugs.

## Background

Communities across North America are experiencing opioid overdose epidemics [1–4]. These epidemics have been largely driven by illicitly manufactured fentanyl, infiltrating the illegal market drug supply across the continent [5, 6]. This has catalyzed public health agencies in implementing a variety of programs and services to address the harms associated with illicit substance use. These have included naloxone (i.e., an opioid antagonist used to counter the effects of opioid overdoses) training and distribution programs [1, 7]; drug checking services where, via various technologies, people who use drugs (PWUD) can check the contents of their drugs for contaminants such as illicitly manufactured fentanyl [8, 9]; and, in some jurisdictions, low-barrier supervised consumption sites (SCS), including women-only SCS [10], where people can use drugs under the supervision of trained staff who can respond to overdoses [11, 12]. Despite these life-saving interventions, there remains a need for other targeted strategies to respond to this public health crisis, particularly for those disproportionately affected by overdose death, including those who use drugs alone in housing settings [13] as well as other vulnerable groups, such as Indigenous women [14].

In 2018, in the Canadian province of British Columbia (BC), 86% of overdose deaths occurred indoors (e.g., private housing, social housing, hotels, emergency shelters) [15], making targeted housing-based public health interventions a priority [13]. These numbers remain unchanged in 2019 [16]. In response, a variety of housing-based interventions have been implemented in an attempt to address these preventable deaths. These include: tenant-led naloxone training and distribution programs embedded within single room occupancy (SRO) hotels [17]; housing-based overdose prevention sites, including interventions targeted at women specifically [18]; and peer-witnessed consumption programs within emergency shelters [19]. While these have been effective in responding to, and reversing, opioid-related overdoses, individuals using contaminated drugs alone remain most at risk of overdose death [15], which lessens the likelihood that another person could respond with naloxone and call emergency services. Thus, under the restraints of prohibition, additional overdose prevention approaches are required in order to address those who are most vulnerable to overdose fatalities.

Studies have demonstrated a variety of reasons why people use drugs alone. Some studies have suggested that PWUD experience shame and stigma regarding their drug use and therefore use alone as a way to hide their use [19–22]. Sharing drugs with others is a common practice so some use alone to avoid this practice [19, 23]. Other research has demonstrated how policing and the criminalization of drugs and the people who use them leads individuals to hide their use to avoid police harassment or arrest [24] and some are unaware or skeptical of Good Samaritan laws that may protect them from arrest [22, 25]. One recent study indicated that some women use drugs alone as a means to prevent intimate partner violence [18]. Given that there is a plethora of reasons why people use drugs alone, interventions that address overdose risk among this cohort of PWUD are urgently needed.

The experiences of PWUD in our study setting are framed by their structural vulnerability. For the purpose of this article, structural vulnerability can be defined as the multiple vulnerabilities that particular groups of PWUD (e.g., Indigenous people, sex workers, people living in poverty) experience because of their marginalized positions within hierarchical structures (e.g., colonialism, patriarchy, capitalism) [26]. Macro level social and structural contexts increase the structural vulnerabilities of some communities of PWUD. Contextual factors, such as poverty, criminalization, and gender- and racial-based violence increase vulnerabilities across oppressed groups and negatively impact their health as well as social and economic well-being [27, 28].

Research on women who use drugs, specifically, has identified a variety of determinants that increase their structural vulnerability. These include experiences of physical and sexual violence [29], increased risk of hepatitis C [30] and HIV transmission [31], and for Indigenous women, discrimination, colonization, and systemic poverty [32]. In BC, for example, women who use criminalized drugs and are also engaged in sex work can experience increased risk of harm (e.g., vulnerability to overdose and violence) due to the criminalization and stigma of substance use and sex work, with Indigenous sex workers among those most vulnerable [25, 33–35]. Indigenous women in BC are also disproportionately affected by overdose death [14], with the overdose death rate for First Nations women in 2019 8.7 times the rate for non-First Nations women [36].

These structural vulnerabilities shape the day-to-day experiences of many women who use drugs residing in Vancouver, BC’s Downtown Eastside. This neighbourhood is one of Canada’s epicenters of the overdose crisis and is framed as a site of poverty, homelessness, visible drug use, criminalization, and also community activism [37, 38]. The neighborhood is comprised of many SRO hotels, which are one of the few affordable housing options accessible to people living in poverty. These buildings consist of small individual bedrooms with shared washrooms and showers with conditions that have been described as crowded, unsafe, and unsanitary and negatively impacting the health of residents [17, 39–41]. To address these social and structural inequities, a variety of health and social services also exist in this neighbourhood to provide safer environment interventions [42], some of which are targeted toward women who use drugs, including supportive housing models embedded within SROs and overdose prevention sites [10, 39].

Overdose response mobile applications that target those who use drugs alone within housing environments are emerging harm reduction interventions that warrant further investigation. A variety of technology companies have developed software applications for cellular phones whereby PWUD can alert help via their mobile phones. For example, one application allows the user to enter their exact location in the application and initiate a timer. If the person does not interact with the application when the timer goes off, emergency services are immediately called [43]. However, a study among patients enrolled in drug treatment in the UK found that only 57% owned smartphones, which could limit the effectiveness of such an application [44]. For those who own cellphones, a recent mixed-methods study on the acceptability of smartphone applications for overdose monitoring found that 68% of PWUD who own a cellphone with internet access would utilize applications [45]. However, this study also found that these applications may not benefit PWUD who are transient and those who do not have regular access to mobile devices. For PWUD who are living in poverty and do not have consistent access to working smartphones with internet access, these applications sound less promising.

In BC, a housing organization has implemented a variety of interventions within their low-income supportive housing buildings, including SROs, to address overdoses (e.g., SCS, peer witnessing), which are explicitly designed to be accessible to structurally vulnerable PWUD. Included among these interventions is a wireless overdose response button system. This system allows residents who are using drugs alone to press a wall-mounted battery-powered button (about 1 inch in diameter) prior to their drug use, which then sends a notification to a cellular phone monitored by building support staff who can then check on residents and respond accordingly. The button can only be used in their designated units, as each is assigned a room number. While this type of system may sound more promising for PWUD who do not have smartphones nor internet access, no known research has examined its impact on drug use and overdose response. In this article, we examine the experiences of women residents utilizing this technology within a women-only supportive housing environment.

## Methods

This study draws on qualitative research methods [46] as a means to gain insight into the day-to-day experiences of participants as they related to drug use, overdose risk and prevention, and other socio-structural dimensions of vulnerability. Via interviews, qualitative research aims to provide participants a ‘voice,’ more often absent in quantitative research methods, and is thus a valuable methodological tool when working with structurally vulnerable communities [47]. Data collection occurred in October 2019 at a women-only supportive housing building in Vancouver, Canada. At the time of data collection, the button system had been operating in this building since 2018. Eligibility criteria included currently residing at the supportive housing building and an ability to complete an interview in English.

A qualitative interview guide was first developed by our peer research assistants (i.e., community members trained in research activities with lived expertise of drug use) and then refined in consultation with a group of women with lived experience of drug use. Interview participants were purposively recruited with the help of on-site supportive housing staff and management who then contacted the lead author to schedule interviews. Potential participants were also approached by our research team in common areas of the building. Interviews were completed by GB (postdoctoral fellow; cisman) and TF (Ph.D. student; ciswoman), who have extensive training in community-based qualitative research as well as established relationships with community members and organizations in our study setting. Before each interview, interviewers introduced themselves, provided study information and rationale for doing the research, and an opportunity for participants to ask any questions prior to providing written informed consent. Fourteen semi-structured in-depth interviews were conducted. Interviews took place in an on-site private room or at our research office. Participants were provided an option to have a peer research assistant involved in the interviews. A woman-identified peer research assistant, who had pre-established relationships with many of the study participants, aided in recruitment and co-led some of the interviews in an effort to minimize power relations and foster rapport [48]. Interviews were approximately 15–60 min in length. Participants were provided with $30 (CAD) cash honoraria. The interviews were audio recorded and professionally transcribed. Data collection ended once no other potential participants expressed interest in participating in the study. Despite the relatively small sample size compared to other qualitative studies, the building only housed 22 women at the time of the study, and some residents were unable to participate due to language barriers, disinterest, or health issues (e.g., hospitalization). Moreover, a recent study on data saturation in qualitative research found that a larger sample size has almost no effect on study outcomes, and as few as seven interviews are able to capture the major themes [49]. Importantly, the themes discussed herein were recurring across the interviews.

In consultation with the senior author, the lead author developed a list of overarching themes. The lead author then read and reread the transcripts and completed line-by-line coding. Informed by a grounded theory approach [50, 51], data was then organized into themes for analysis based on both a priori themes (e.g., technology usability, overdose risk) and those that emerged from the data (e.g., violence, alternate uses). Analysis was further informed by theories of structural vulnerability [26–28] as well as feminist perspectives, which seek to identify interlocking systems of oppression and inequity [52, 53] and how these negatively impact the lives of women who use drugs [54]. The University of British Columbia/Providence Health Care Research Ethics Board granted ethical approval for this study.

## Results

The study sample included interviews with 14 cisgender women (including one who also identified as Two-Spirit). Nine participants were Indigenous, three identified as white, one was racialized non-Indigenous, and one undisclosed. Participant narratives framed their structural vulnerability [26]. Almost all of the participants reported poly-substance use (i.e., used both opioids and stimulants) and the majority used drugs daily. All but one participant disclosed active engagement in sex work. All participants were on social assistance and living in poverty. See Table 1 for further demographic information. Given the small size of the building, in order to protect the anonymity of participants, we chose not to include specific demographics after each quote. Below are some notable themes from our findings.

**Table 1:** Sample characteristics (n = 14)

**Age**	
Range	30–55
Average (mean)	39
Undisclosed	2
**Gender**	
Cis woman	14
Two-spirited	1
**Race/ethnicity**	
Indigenous	9
White	3
Racialized/non-indigenous	1
Undisclosed	1
**Current substances used**	
Opioids and stimulants	11
Stimulants only	2
Undisclosed	1
**Frequency of use**	
Daily	12
3–4 times per week	1
Undisclosed	1
**Experienced an overdose**	
In the last year	7
Undisclosed	1
**Income from sex work**	
Last 30 days	13

### Drug use context

We asked participants to describe the overdose context of their building *prior* to the implementation of the overdose response technology. Participants noted they frequently used drugs alone, but they also described using with, or in the presence of, partners, friends, and sex work clients. Many participants described the challenges of responding to frequent overdoses in a three-story SRO that, at the time of the study, housed 22 women. Getting staff support in the event of an overdose was discussed by some participants as onerous and requiring significant time, as illustrated in the following quotes: *“I’d just scream and bang on the door. You know, thump on the door because right underneath me is the front desk, right?”* (Participant 13), and *“We had to run to the top of the stairs to yell for staff…We’d get somebody to go down the stairs and get the staff”* (Participant 3).

Leaving one’s room to go and retrieve staff was not only described as onerous, but also involved leaving someone who was overdosing behind and potentially alone without support. For example:

*There’s been times that I needed the staff to come there and stuff and I didn’t know how to get them to get up there without me leaving my room… Before I had to run downstairs and get some of the staff to come up and you’re scared about that person during that time, right?* (Participant 14)

An emergency button was described as a more efficient way to get assistance from staff.

### Overdose response

Participants provided varied responses when asked about their use of the overdose response button. Most participants indicated that they did not use the button in its intended way (i.e., pressing it before consuming drugs to alert staff to check on them). Only one participant described using the button as intended, and only when she used alone:

*If I am using [drugs] upstairs in my room by myself, I always just push it once just to have them* [i.e., building staff] *come check up on me…I haven’t had any bad experiences, like emergency situations, but I just use it just for checkups…the staff are pretty quick to come upstairs and check up on people.* (Participant 4)

Another participant described using the button before she overdosed, though she did not press it prior to her use, but rather, during:

*I had picked up some crystal [methamphetamine], and I had about three grams. I smoked some, I did a shot* [i.e., injection], *and then when I was going back to smoke the last of it…and I started shaking right away. I was sweating and I couldn’t move. But I forced my leg…like that’s when I used my toe for the button. And the staff came upstairs and said ‘I’ve got to Narcan you.’ And that’s all I remember. Five hours later I woke up at [the hospital].* (Participant 3)

This quote not only demonstrates how the participant experienced physical cues of an overdose and was able to press the button before she lost consciousness, but also demonstrates a different perception of overdose risk whereby the technology was not used as intended.

Most participants indicated that they did not regularly press the button for drug use—even when they were using alone—with one participant commenting that she never uses it. For example, when asked if the button is pressed every time drugs are used, one participant described relying on her own instincts and contacts rather than the button: *“Oh no. No, you know, like I said, I’m pretty responsible. I know what I’m using…and if the dope changes, my people tell me”* (Participant 7). Other participants spoke about forgetting to use it altogether. When asked about her frequency using the button, one participant exclaimed: *“Not that often, no. Not when I’m by myself. I always forget. I’ve used it maybe a couple of times but not that often”* (Participant 14). Another participant described sporadic use, with specific instances when she forgets to use it:

*Usually if I’m too fucked up* [i.e., intoxicated]*, I don’t think. I don’t really think about it. But if…like if I went out and scored* [i.e., bought drugs] *and came home, and have come upstairs, seen the staff, then it’s fresh in my mind, like, ‘oh yeah, I should press the button’ so then – then I will.* (Participant 5)

Levels of impairment may impact use of an overdose response intervention, and consequently, increase one’s vulnerability to overdose risk.

Aside from participant experiences using the buttons for their individual drug use in varying ways, participants more often spoke about utilizing the button in response to other people overdosing—whether in their rooms or elsewhere in the building. When asked about the last time she pressed the button, one participant said:

*A friend of mine overdosed. I went to the washroom and came back, and he was blue. [I: And you just immediately pressed the button?] Oh, yeah. Yeah. [I: How quickly did the staff come?] Right away…they brought him back to life.* (Participant 6)

### Gender-based violence

Participants indicated that while their use of the button for their personal drug use was not common practice, they did utilize the technology for other emergencies they had, including negative interactions with guests that were framed by gendered dynamics. Almost all participants disclosed engaging in sex work in the last 30 days, and many described using the button in emergency situations related to sex work, including violence, sexual assault, and getting “ripped off.” Some participants discussed exclusively using it for this reason, demonstrating how the technology was redefined based on their actual needs. According to one participant,

*It’s basically for emergency purposes, if a man hurts you, or if the woman gets abused by a man, or physically or verbally abused, or attacked in a way…they could run to the button and press the button and cry for help. And whether they fall or not* [i.e., overdose], *it’s a priority of the staff to get upstairs as fast as they can.* (Participant 12)

Another participant discussed feeling safer with having the button system in place, characterizing it as a luxury, though she noted she had not had to use it for any clients thus far:

*For safety issues it makes it a lot quicker. And it goes to them [staff] right on their cell phone, right. Where they can come to you wherever they are at. It’s like a pager system almost, right, which is like a luxury thing to have. You know, it is. Makes you feel a lot safer that, you know, that you have help there. When I found out they will come and help escort your dates out, right, I just haven’t really needed to use it so much for that.* (Participant 14)

Lastly, some participants described relying on each other for help, but described the button as useful when no one is around to assist. For example:

*I think it’s really important for the girls to have in their rooms because a lot of times there’s bad dates* [i.e., violent clients] *and sometimes people are not gonna hear. You know, usually when we hear anybody hollering, whether we like them or not, or get along with them or not, the girls will always come and check it out and make sure that they’re okay…But sometimes people are sleeping and we’re not always gonna hear, right?* (Participant 7)

These quotes indicate that the button system is used in a variety of ways, providing some participants an increased sense of safety in that staff were able to quickly respond to any emergency, drug-related or not. A couple of participants did report technical concerns regarding the button (e.g., if it breaks or if the battery dies), but had not yet experienced these potential barriers to effectiveness.

## Discussion

Our findings demonstrate the multiple structural vulnerabilities [26–28] experienced by participants as they navigated their daily interpersonal, work, and social lives within their housing environment. Participants generally did not utilize the mobile overdose prevention technology for its intended purpose (i.e., pressing *before* individual drug use). However, they did use it for other emergencies such as sex work-related violence and guests’ or other tenants’ overdoses, highlighting their agency to enact some risk reduction strategies.

While this technology was designed to address overdose risk, our findings demonstrate that it was also used for other productive purposes. Previous research has demonstrated how harm reduction technologies (e.g., injection and smoking equipment, naloxone) have been used differently than public health institutional directives [55–57]. For example, research among people who use crystal methamphetamine has highlighted how pipes designed for smoking crack cocaine are not suitable for smoking crystal methamphetamine. However, some use crack pipes in alternative ways to do “hot rails,” a method whereby someone heats up crystal methamphetamine in the stem of a crack pipe to inhale the drug intranasally [57]. Additionally, recent research on naloxone administration highlights how PWUD have reconfigured its technological use so that it not only functions as an overdose reversal action, but affords possibilities to reduce unpleasant and discomforting experiences when receiving naloxone administration through particular practices of care when administered by a community member rather than an unknown paramedic [55]. Social conditions and relations impact harm reduction technology practices among PWUD, and in our study, affected both how and when participants used the button technology. While participants did not frequently use it as intended, it afforded possibilities in responding to interpersonal gender-based violence.

That participants generally did not use the technology as it was intended highlights the ways that the perception of risk is context-dependent and some risks may be perceived as more threatening than others, leading PWUD to prioritize differing risks [23, 58]. Our findings demonstrate that pressing the button prior to using drugs, as was initially intended, was not perceived as a necessary risk-mitigation strategy, but when risk or harm was perceived as explicit (e.g., when someone else was experiencing an overdose event, when participants experienced violence, or when people in other rooms were yelling for help), participants utilized the button. This also demonstrates women’s ability to enact their agency via risk management strategies that address multiple emergencies beyond the intended purpose of the technology.

Critical health research calls attention to the ways in which social, structural, economic, and physical environments affect varying levels of drug-related risk and harm among PWUD and the need for interventions that address these [26, 42, 59, 60]. Residents in our research setting, including our study participants, not only experience structural vulnerabilities due to poverty and criminalization (of drug use and sex work), but also extremely high rates of everyday gendered and racialized violence, disproportionately impacting Indigenous women and women engaged in sex work [25, 32, 34, 61]. These experiences have led some women to use drugs alone to avoid interpersonal violence, creating greater risk of overdose death [18], particularly among Indigenous women, who are disproportionately affected by BC’s overdose crisis [36]. These larger contextual factors (e.g., the dual criminalization of drugs and sex work, colonial practices and policies) associated with overdose risk and gender-, race- and sex work-based violence, exacerbate risk and harms among women who use drugs in our study in ways that the button intervention cannot address. Rather, the button technology is an individualized response mechanism (rather than a broad preventative measure) that offers some real-time opportunities to mitigate harm. As such, its effectiveness should be considered alongside critical safer environment interventions that are culturally-attentive [62] and which address the intersecting gendered, racialized, and criminalized overdose risk environment.

It has been argued elsewhere that an effective, equitable overdose response needs to take into account the syndemics of both overdose and gendered and racialized violence against women [63]. Violence against sex workers is significant globally [64]. In the Canadian context, sex work (like illicit drug use) remains criminalized, which increases sex worker’s exposure to violence, particularly for structurally vulnerable sex workers living in poverty, including most of our participants [25, 65, 66]. Research on sex workers living in SROs in Vancouver has demonstrated that housing environments can make women more vulnerable to violence and sexual exploitation due to their inhabitable physical environments, leading some women to stay in potentially unsafe locations (e.g., mix gender shelters, couch surfing), and guest curfews leading sex workers to work outdoors where their safety was compromised [39]. However, women-only housing environments can also provide opportunities for safer working conditions [39]. Given the social networks our participants described relying upon within their women-only housing environment for overdose response and sex-work related violence prevention, peer-led support systems, which have been used for overdose prevention elsewhere [67, 68], represent one means to create a safer living/work environment among women sex workers in SRO settings [39]. However, there is also the potential to place too much responsibility on a vulnerable group with limited power and resources [69]. Thus, future interventions must also address both the hidden labour and precarity of peer work among sex workers and PWUD [25, 70] in order to improve their health, economic, and workplace conditions as well as opportunities to enact greater agency.

In comparison to other overdose response technologies that either require PWUD to have a cellular phone with consistent internet access (such as mobile software applications) or require PWUD to leave their homes and access them elsewhere (such as drug checking technologies), there appear to be minimal barriers to utilizing this particular technology specifically within supportive housing, as it is consistently connected to a network and located within participants’ homes where they engage in drug use and sex work. However, there are pre-existing conditions that are required in order for this technology to be introduced into other housing environments beyond supportive housing. These conditions include: costs associated with implementation, ongoing maintenance, and internet connectivity (which may not be accessible to structurally vulnerable PWUD); community buy-in from both residents and building owners/management; and on-site staff dedicated to respond to emergencies. This technology may benefit private housing settings if the intervention was led by tenants; however, given that sex work continues to be criminalized, those who participate in this intervention may be targeted by building management, leading to harassment and possible eviction, which have been identified as concerns among participants of another tenant-led intervention in our study setting [17]. Importantly, the development and implementation of any technological intervention targeting vulnerable communities need to incorporate experiential knowledge on how interventions work, including limitations and alternate uses, to more appropriately conceptualize technologies that speak to the interconnectedness of people’s experiences. This should consist of community-based research methods prior to implementation.

There are limitations to this study. First, while participants were provided with the option to have interviews co-led by a peer research assistant, we are aware that there still may have been power dynamics that influenced what information participants were comfortable with sharing in the interviews. Second, participants’ experiences may not be applicable to tenants in other buildings. Our limited study sample was exclusively women, and does not address how men or people living in mixed gender buildings utilize the technology. Thus, future research should examine the ways in which overdose prevention interventions are utilized based on gender and other social determinants [71, 72]. Lastly, we were prevented from conducting additional research on this intervention in other buildings when it was discovered that the button technology company was providing incentives (e.g., company shares) to residents, which raised ethical concerns for ongoing research based on competing interests potentially impacting study findings. However, we believe that the findings in this article are not compromised as drug use, overdose risk, and the violence experienced by women and sex workers is well-documented in our study setting [10, 25, 29, 39, 63] and our findings illustrate alternative ways that women adapted this technology for other emergencies rather than its intended purposes (i.e., pressing before consuming drugs). Further studies are needed that also include the perspectives of other stakeholders, such as building staff and management, to further characterize overdose response as well as any other technological limitations not described herein.

## Conclusions

In conclusion, our findings demonstrate the clear need for housing-based emergency response interventions that address not just overdose risk for those who use drugs alone but also for gender-based violence. While mobile applications and technologies offer some real-time possibilities for risk mitigation, larger socio-structural changes remain critical to reducing the structural vulnerabilities experienced by women who use drugs, sex workers, and Indigenous people. These include the decriminalization of drugs and sex work and providing a regulated supply of drugs to PWUD to replace the toxic illegal drug supply responsible for the majority of overdose deaths in BC and elsewhere. In the meantime, immediate housing-based interventions are needed to lessen the negative consequences of criminalization and a toxic drug supply, but these must be implemented as part of a larger strategy to address the structural issues that are impacting marginalized communities in detrimental ways, including poverty, overdose, and violence.

## Data Availability

The qualitative datasets for this study are not publicly available given the sensitive nature of the topic, as they contain confidential information that could compromise participant confidentiality and consent. Additionally, the interview guide is not available at this time due to its ongoing use as part of a longitudinal study. However, it may be available in the future on reasonable request via the lead author.

## Abbreviations

BC: British Columbia
PWUD: People who use drugs
SCS: Supervised consumption sites
